# Oral microbiome dysbiosis in cryptogenic ischemic stroke patients with high-risk patent foramen ovale

**DOI:** 10.1038/s41598-025-95728-x

**Published:** 2025-04-04

**Authors:** Muhammed Manzoor, Jaakko Leskelä, Milla Pietiäinen, Nicolas Martinez-Majander, Eija Könönen, Juha Sinisalo, Jukka Putaala, Pirkko J. Pussinen, Susanna Paju

**Affiliations:** 1 Department of Oral and Maxillofacial Diseases, University of Helsinki, PO Box 63, 00014 Helsinki, Finland; 2 Sustainable Products and Materials, VTT Technical Research Centre of Finland, 02150 Espoo, Finland; 3 Department of Neurology, Helsinki University Hospital, University of Helsinki, 00290 Helsinki, Finland; 4 Institute of Dentistry, University of Turku, 20500 Turku, Finland; 5 Heart and Lung Center, Helsinki University Central Hospital, Helsinki University, 00260 Helsinki, Finland; 6 School of Medicine, Institute of Dentistry, University of Eastern Finland, 70211 Kuopio, Finland

**Keywords:** Cryptogenic ischemic stroke, Oral microbiome, Patent foramen ovale, Stroke, Paradoxical embolism, Saliva, Metagenomic sequencing, Cardiovascular diseases, Microbial communities

## Abstract

Patent foramen ovale (PFO) is the most common congenital heart abnormality of foetal origin and has been associated with cryptogenic ischemic stroke (CIS) through several mechanisms, with most theories supporting paradoxical embolism. Other possible but unknown contributing factors, such as the role of the microbiome in PFO-associated strokes, remain unclear. We analysed saliva metagenomes to study the differences in the oral microbiome between young-onset CIS patients with clinically relevant high-risk PFO (n = 52) and those without PFO (n = 52). Age- and sex-matched stroke-free controls (n = 16) with high-risk PFO were included for the comparison. Beta diversity was significantly different between patients and controls with high-risk PFO, but not between patients with and without high-risk PFO. The phylum *Ascomycota* and class *Saccharomycetes* were significantly more abundant in patients with high-risk PFO than in those without high-risk PFO. Additionally, the abundance of *Lactococcus*, including *Lactococcus raffinolactis* and *L. cremoris*, was higher in controls with high-risk PFO than in patients with high-risk PFO. These findings highlight that oral dysbiosis and high-risk PFO may form a critical but under-recognized combination in the aetiology of CIS. Future research should focus on elucidating the precise mechanisms of these interactions and developing targeted interventions.

**Supplementary Information:**

The online version contains supplementary material available at 10.1038/s41598-025-95728-x.

## Introduction

Patent foramen ovale (PFO) is the most common congenital cardiac anomaly of foetal origin and is characterized by incomplete closure of the atrial septum between the right and left atria^1^. Approximately 25–30% of the adult population have PFO, whereas the prevalence of PFO is 40–50% in patients diagnosed with cryptogenic ischemic stroke (CIS)^1,2^. PFO is implicated in paradoxical embolism, where venous thrombi bypass pulmonary circulation and enter the systemic arterial system, significantly increasing the risk of cerebral ischemic events^3^. Current understanding supports the idea that PFOs, in particular with certain high-risk features, such as large-sized shunt and associated atrial septal aneurysm, carry the highest causality and are clinically relevant, i.e. often referred to transcatheter closure if detected after CIS^4^.

The incidence of ischemic stroke among young adults is rising globally^5^, particularly those with no apparent aetiology (cryptogenic), and poses significant clinical challenges because of its multifactorial nature and frequent indeterminate origins^6^. The risk factors for stroke in young adults closely mirror those observed in the general population, with predominant modifiable factors including hypertension, smoking, obesity, and diabetes mellitus^7,8^. However, behavioural risk factors are more common among younger individuals and several risk factors specific to young age have been identified, including pregnancy, puerperium, heavy alcohol consumption, and illicit drug use^9^.

Recent research has elucidated the potential impact of oral microbiome dysbiosis on various systemic conditions, such as cardiovascular and neurological outcomes and stroke^10–12^, through mechanisms involving systemic inflammation, immune modulation, and direct bacterial translocation. In our previous study, we observed that dysbiosis of the oral microbiota was associated with CIS in young adults and identified a series of oral markers, including bacterial, viral, and fungal species associated with CIS^13^.

Oral microbiota and their products can enter the bloodstream, potentially contributing to endothelial dysfunction, thrombosis, and atherogenesis^14^, which are significant factors in CIS patients. Similarly, in patients with PFO, right-to-left shunting can facilitate the direct passage of oral microbiota from venous circulation into arterial circulation^15^. Consequently, these microbial emboli (clusters of microbes or infected material) can travel to the brain, occluding the cerebral vessels and leading to ischemic stroke. Bacteraemia is frequent during invasive dental treatments and can also occur during routine oral hygiene practices, such as tooth brushing^16^, thus potentially increasing the risk of acute cardiovascular events^17^. Indeed, in our previous study, prior invasive dental treatment was associated with an increased risk of CIS^18^. Furthermore, this association was especially strong among patients with PFO.

Despite established connections between PFO and CIS^19^, the specific role of the oral microbiome in CIS patients with high-risk PFO remains poorly understood. To our knowledge, no study has directly investigated the relationship between oral microbiome dysbiosis and PFO-associated stroke risk. We hypothesized that (1) the oral microbiome in CIS patients with high-risk PFO differs from that in patients without PFO, and (2) the oral microbiome in CIS patients with high-risk PFO differs from that in controls with high-risk PFO. To investigate this hypothesis, we utilized high-throughput shotgun metagenomic sequencing of saliva to identify the diversity and composition of microbiota linked to high-risk PFO in young patients with CIS.

## Methods

### Study design

The present study, SECRETO Oral, is a substudy of the international, multicenter SECRETO study (Searching for Explanations for Cryptogenic Stroke in the Young: Revealing the Etiology, Triggers, and Outcome, NCT01934725)^20^. The SECRETO Oral cohort included data from 329 participants (169 cases and 160 controls) ^13,18^. For this study, we included CIS patients with high-risk PFO, CIS patients without PFO, and stroke-free controls with high-risk PFO, excluding participants who had used antibiotics within one-month preceding saliva sample collection, as well as those without information on antibiotic use history. Participants were recruited in this study between December 2013 and November 2019 at Helsinki University Hospital and Turku University Central Hospital.

We screened consecutive young adults aged 18–49 years with first-ever imaging-positive acute ischemic stroke, diagnosed by neurologists in the participating centres. All patients underwent brain magnetic resonance imaging (MRI), angiography of intracranial and extracranial vessels, echocardiography, and screening for coagulopathies, and laboratory testing per protocol. One sex-, age- (± 5 years), and ethnicity-matched stroke-free control for each patient from the same region was sought locally at each study centre.

CIS was then defined according to the Atherosclerosis, Small vessel disease, cardiac source, and other causes (A-S-C-O) classification^21^, with minor adjustments to better capture the clinical uncertainties typically observed in younger patient studies^20^. We included all patients with PFO to capture the full range of PFO-related stroke, as knowledge of factors that increase PFO causality has been accruing over time and the inaugural mechanism of thrombosis in PFO-associated strokes remain poorly studied. All strokes were acute, and chronic stroke was excluded. Stroke severity was evaluated using the National Institutes of Health Stroke Scale (NIHSS) score (range 0–42, with higher scores indicating greater disease severity), and was classified as: (1) 0 (no scorable symptoms on admission), (2) 1–4 (mild symptoms) (3), 5–9 (moderate), and (4) ≥ 10 (severe).

### Cardiac and aortic examinations

The participants underwent transthoracic echocardiography (TTE) and transesophageal echocardiography (TEE) to assess the cardiac structure and function. The ejection fraction was calculated from the echocardiographic data. Additionally, transcranial Doppler ultrasound bubble tests (TCD-BS) were conducted to identify right-to-left shunts. The maximum volume of the interatrial and right-to-left shunts was classified as small, moderate, or severe based on the bubble count. Atherosclerosis assessments of the ascending aorta and the aortic arch were also performed.

PFO was diagnosed with colour Doppler imaging showing spontaneous right-to-left shunt or shift of shunt direction using Valsalva manoeuvre. Confirmation of PFO required a bubble study with microbubbles appearing in the left atrium during the first 3–5 cardiac cycles in TEE. In most participants, an additional TCD-BS with Valsalva manoeuvre was performed to quantify the right-to-left shunts. All available information from TTE, TEE, and TCD bubble studies were used to identify and define high-risk PFO. Clinically relevant high-risk PFO was then defined as PFO with an atrial septal aneurysm or a large-sized shunt (≥ 25 microbubbles crossing the atrial septum in TEE or detected in transcranial Doppler).

### Cardiovascular risk factors and clinical oral examination

The participants underwent a thorough structured interview at the time of recruitment and a detailed clinical history was obtained from all participants using medical records. Body mass index (BMI) was determined by measuring the participants’ weight and height. Data on antibiotic use were collected from medical records. The registered cardiovascular risk factors included hypertension, abdominal obesity, current or former tobacco smoking, and heavy alcohol consumption^20^.

The same periodontal specialist (SP) conducted the clinical oral examinations in a standard dental office setting between April 2014 and February 2020. During the examinations, bleeding on probing (BOP) was recorded at six sites per tooth and the number of missing teeth was documented. Regular dentist check-ups were also documented. Caries lesions were diagnosed in clinical settings using the complete International Caries Detection and Assessment System (ICDAS) code. Periodontitis staging and grading were also performed^18,22^ and mucosal lesions and changes were recorded.

### Saliva sample processing and metagenomic analysis

Details on saliva sample collection, processing, DNA extraction, and metagenomic sequencing have been previously described^13,23^. In brief, saliva samples were mixed with lysis buffer and subjected to repeated bead beating for DNA extraction. DNA extraction followed the protocols of the ChemagicTM 360 instrument (PerkinElmer). DNA libraries were prepared using the NEBNext® Ultra™ II FS DNA Library preparation kit. Paired-end shotgun metagenomic sequencing was conducted on an Illumina NovaSeq 6000 (San Diego, CA, USA). Subsequent preprocessing of sequence data was performed using FastQC (v. 0.11.9), MultiQC (v. 1.9), and Trimmomatic (v. 0.39). Host-associated reads were removed using Kneaddata with the human genome (GRCh38.p14). Taxonomy profiling was performed using Kraken2 (v. 2.1.2)^24^. Bracken (v. 2.7) was used to refine the estimates of species-level abundance^25^.

### Statistical analysis

Statistical analyses were performed using IBM SPSS Statistics (version 29.0.2.0, IBM Corp.). The analyses included Mann–Whitney *U* test, Kruskal–Wallis test, and independent samples t-test, depending on the data type. All microbiome analyses were performed in R (v. 4.3.1). The sequence data, along with the metadata, were stored in specialized data containers designed for microbiome research in R, namely Phyloseq (v. 1.44.0) and TreeSummarized Experiment (v. 2.10.0) for further analysis. Before analysis, we removed sequences with fewer than 100 reads. We also excluded taxa present in < 10% of the samples. For analysis, taxonomic read counts were normalized to relative abundances and transformed using a centered log-ratio (CLR) method for downstream statistical analyses.

Alpha diversity was estimated using the Shannon and Inverse Simpson indices. Differences in community composition (beta diversity) were determined by permutational multivariate ANOVA (PERMANOVA) using the vegan package (v. 2.6.4)^26^ with the adonis2 function. Principal Coordinate Analysis (PCoA) was performed based on both Bray–Curtis dissimilarity and Jaccard distance to visualize the microbiome structure. We used the DESeq2 (v1.38.2) to identify differentially abundant microbiome features between the groups^27^. *P*-values were corrected for multiple testing using the Benjamini–Hochberg procedure, with a significance threshold set at *q* ≤ *0.05*.

## Results

### Clinical characteristics

A total of 120 participants (52 patients with high-risk PFO, 52 patients without PFO, and 16 controls with high-risk PFO) were included in this study. Mean age (SD) of participants was 40.6 (8.1) years.

The clinical characteristics of the patients with and without high-risk PFO are summarized in Table 1. The difference in sex distribution between the two groups was not statistically significant (*p* = 0.158). Patients with high-risk PFO had a significantly higher mean age (41.01 ± 6.5 years) than patients without PFO (36.98 ± 9.2 years) (*p* = 0.040). Heavy alcohol use was reported by 13.5% of patients with high-risk PFO and 42.3% patients without high-risk PFO (*p* = 0.001). Additionally, 13.5% of the patients used antibiotics in the preceding 3 months (17.3% of patients with high-risk PFO and 9.6% of patients without high-risk PFO, *p* = 0.253). Dentine caries lesions were present in 48.1% of the patients (*p* = 0.118), periodontitis in 26.9% (*p* = 0.206), and mucosal lesions in 19.2% (*p* = 0.150). None of these conditions showed a significant difference between patients with and without high-risk PFO. However, a significantly higher proportion of patients without PFO had hypertension (32.7%) than those with high-risk PFO (3.8%) (*p* < 0.001). The distribution of stroke severity showed no significant difference between patients with high-risk PFO and those without PFO (*p* = 0.413, Table 1).

**Table 1:** Characteristics of included CIS patients with high-risk PFO and without PFO.

		Patients with high-risk PFO (n = 52)	Patients without PFO (n = 52)	*p-*value
Gender	Male	36 (69.2)	29 (55.8)	0.158
	Female	16 (30.8)	23 (44.2)	
Mean age (SD), years		41.01 (6.5)	36.98 (9.2)	**0.040**
BMI (SD), kg/m^2^		26.8 (4.1)	26.3 (4.4)	0.266
Smoking ever	Yes	21 (40.4)	30 (57.7)	0.079
	No	31 (59.6)	22 (42.3)	
Heavy alcohol use	Yes	7 (13.5)	22 (42.3)	**0.001**
	No	45 (86.5)	30 (57.7)	
Abdominal obesity	Yes	25 (48.1)	25 (48.1)	1.000
	No	27 (51.9)	27 (51.9)	
Hypertension	Yes	2 (3.8)	17 (32.7)	** < 0.001**
	No	50 (96.2)	35 (67.3)	
Antibiotics (preceding 3 months)	Yes	9 (17.3)	5 (9.6)	0.253
	No	43 (82.7)	47 (90.4)	
Caries	Yes	21 (40.4)	29 (55.8)	0.118
	No	31 (59.6)	23 (44.2)	
Periodontitis^#^	Yes	11 (21.2)	17 (32.7)	0.206
	No	39 (75)	34 (65.4)	
Mucosal lesions and changes*	Yes	13 (25)	7 (13.5)	0.150
	No	39 (75)	44 (84.6)	
Regular dentist check-ups^†^	Yes	27 (51.9)	26 (50.0)	0.924
	No	25 (48.1)	25 (48.1)	
Bleeding on probing, mean (SD)		41.1 (14.9)	43.1 (14.4)	0.742
Number of missing teeth				0.952
0		36 (69.2)	36 (69.2)	
1–2		6 (11.5)	7 (13.5)	
3–5		9 (17.3)	8 (15.4)	
> 5		1 (1.9)	1 (1.9)	
Stroke severity on admission, NIHSS score*				0.413
No symptoms		13 (25)	14 (26.9)	
Mild		32 (61.5)	24 (46.2)	
Moderate		6 (11.5)	12 (23.1)	
Severe		1 (1.9)	2 (3.8)	

BMI, Body mass index; SD, standard deviation; Abdominal obesity; men ≥ 0.9, women ≥ 0.85; *NIHSS score: 0 (no scorable symptoms on admission), 1–4 (mild symptoms), 5–9 (moderate symptoms), and ≥ 10 (severe symptoms). Missing data: ^#^periodontitis n = 3, ^†^ regular dentist check-ups n = 1, * Mucosal lesions and changes n = 1.

Significant values are in bold.

There was no significant difference in ejection fraction between patients with high-risk PFO and those without PFO (*p* = 0.315). Atrial septal aneurysm was found in 11.5% of patients with high-risk PFO; none was found in patients without PFO (*p* = 0.011) (Table 2).

**Table 2:** Cardiac and aortic examinations and findings among the included patients with high-risk PFO and without PFO.

		Patients with high-risk PFO (n = 52)	Patients without PFO (n = 52)	*p-*value
Transthoracic echocardiography performed	Yes	52 (100)	52 (100)	1.000
	No	0	0	
Transesophageal echocardiography performed	Yes	51 (98.1)	49 (94.2)	0.310
	No	1 (1.9)	3 (5.8)	
TCD-BS performed	Yes	50 (96.2)	43 (82.7)	**0.026**
	No	2 (3.8)	9 (17.3)	
Ejection fraction (%)		64.6 (7.6)	63.9 (6.9)	0.315
PFO found by echocardiography	Yes	46 (88.5)	0	** < 0.001**
	No	6 (11.5)	52 (100)	
Right-to-left shunt (PFO) found by TCD-BS^$^	Yes	48 (92.3)	0	** < 0.001**
	No	2 (3.8)	43 (82.7)	
Maximum volume of interatrial shunt in echo*				–
Small		6 (11.5)	–	
Moderate		10 (19.2)	–	
Severe		30 (57.7)	–	
Maximum volume of right-to- left shunt in TCD-BS*^#^				–
Small		4 (7.7)	–	
Moderate		2 (3.8)	–	
Severe		42 (80.8)	–	
Atrial septal defect	Yes	1 (1.9)	1 (1.9)	1.000
	No	51 (98.1)	51 (98.1)	
Atrial septal aneurysm^#^	Yes	6 (11.5)	0	**0.011**
	No	45 (86.5)	52 (100)	
Atherosclerosis in ascending aorta^µ^	Yes	–	1 (1.9)	0.307
	No	50 (96.2)	47 (90.4)	
Atherosclerosis in aortic arch^†^	Yes	–	–	1.000
	No	48 (92.3)	45 (86.5)	
Aortic atherosclerosis^£^	Yes			0.307
	No	50 (96.2)	47 (90.4)	

PFO, patent foramen ovale; TCD-BS, transcranial Doppler ultrasound bubble test. *Maximum volume of interatrial right-to-left shunt in echocardiography: small 1–9 bubbles, moderate: 10–24 bubbles, severe: ≥ 25 bubbles.

Significant values are in bold.

### Oral microbiome composition

**Fig. 1:**
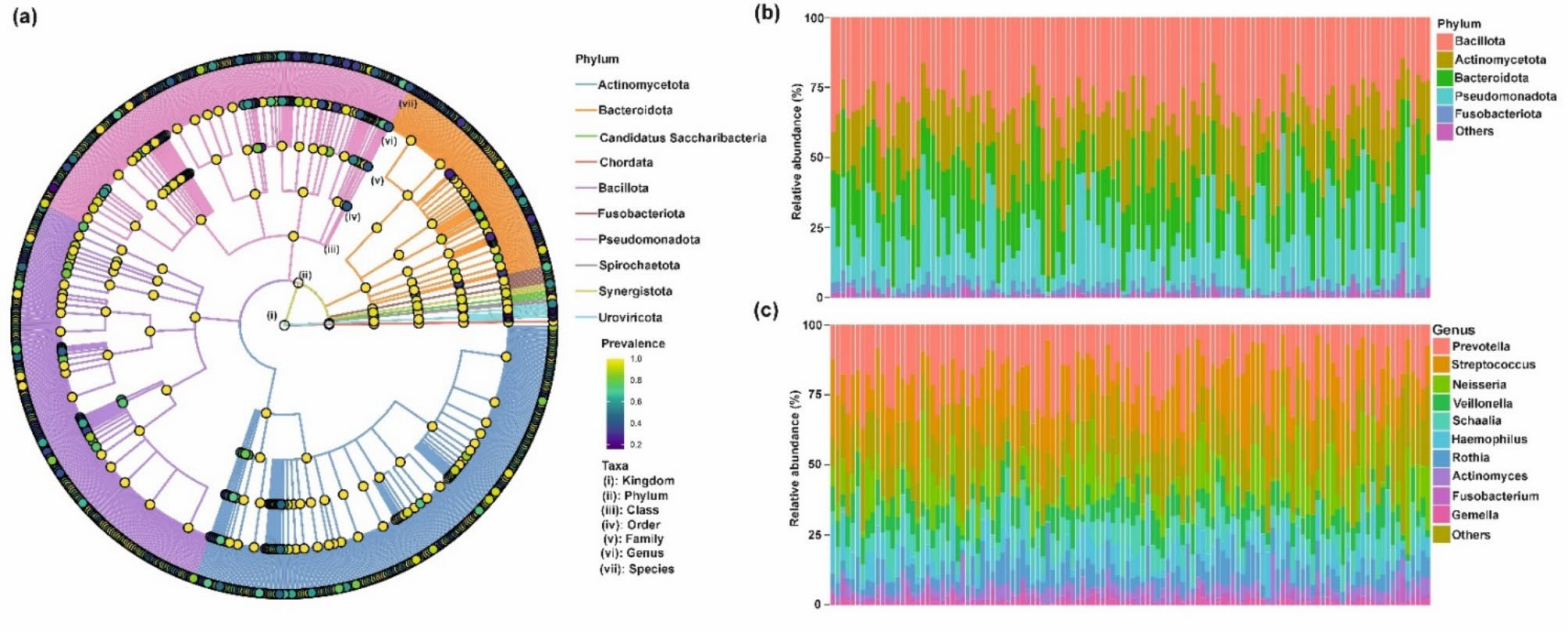
Composition and taxonomic distribution of the oral microbiome. (**a**) Taxonomic tree of the oral microbiome showing the prevalence of top 10 phyla as determined by mean abundance. (**b**) Stacked bars of relative abundances of the five most abundant phyla for all samples, shown in descending order of total abundance. (**c**) Stacked bars of relative abundances of the 10 most abundant genera for all samples, displayed in descending order of total abundance. Genera not included in the top 10 are grouped into the category ‘Others’.

Individual oral microbiome diversity and composition were characterized by metagenomic sequencing of saliva samples. We focused on microbial taxa whose relative abundance exceeded 0.001% (detection) in at least 10% (prevalence) of the samples, which yielded 26 phyla, 53 classes, 115 orders, 243 families, 721 genera, and 2374 species. A taxonomic tree was constructed for the 10 most abundant phyla to illustrate the composition and abundance of the taxonomy (Fig. 1a). Oral microbiota was dominated by the phyla *Bacillota, Actinomycetota, Bacteroidota*, *Pseudomonadota,* and *Fusobacteriota* (Fig. 1b). At the genus level, the most abundant genera in the samples were *Prevotella*, *Streptococcus*, *Neisseria, Veillonella*, *Schaalia* and *Haemophilus* (Fig. 1c).

### Microbiome diversity and composition in CIS patients with and without high-risk PFO

**Fig. 2:**
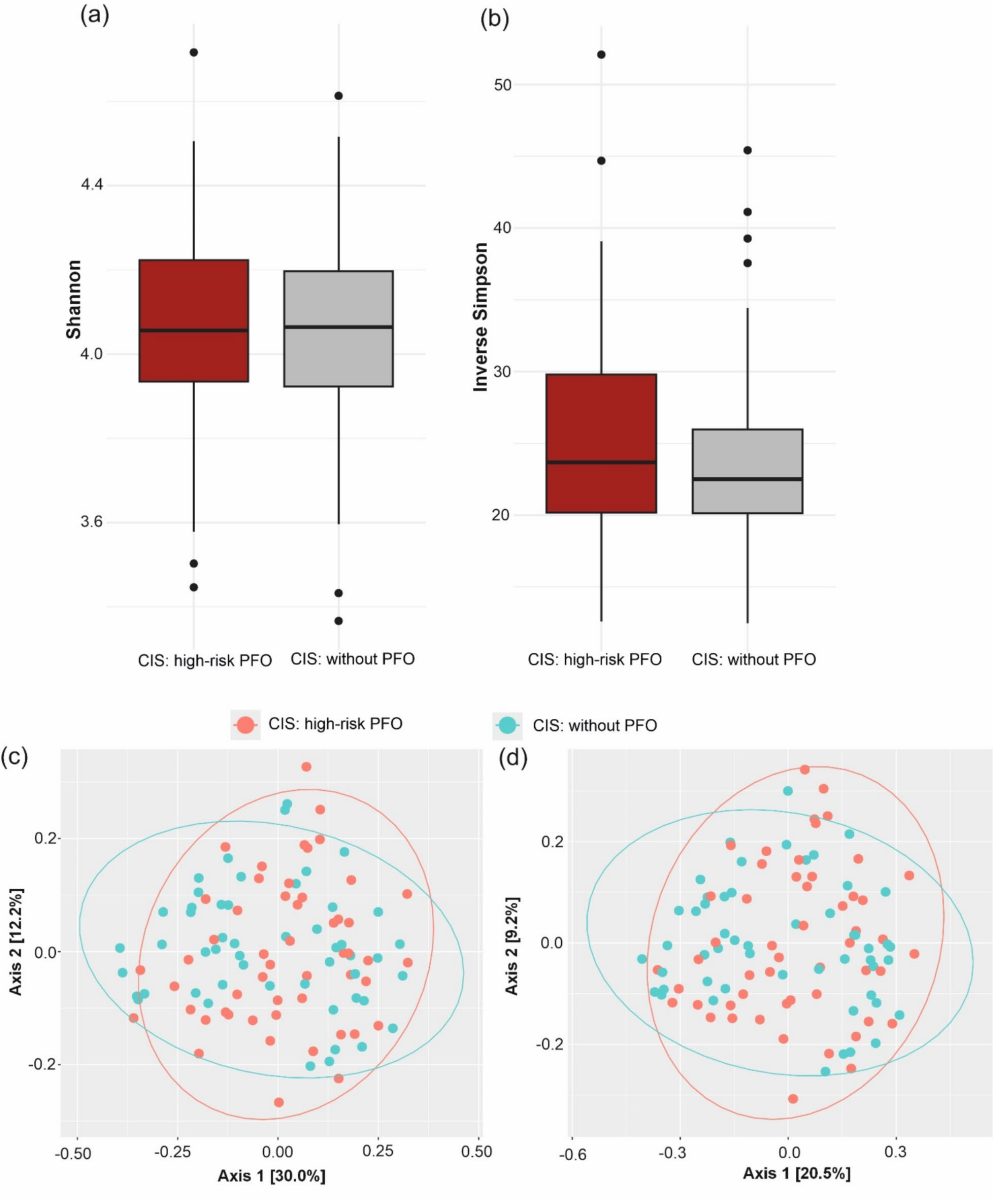
Oral microbiome diversity in CIS patients with and without high-risk PFO. Alpha diversity measurements using the (**a**) Shannon and (**b**) Inverse Simpson index between CIS patients with high-risk PFO and without PFO. PCoA plot of beta diversity of the oral microbiome based on (**c**) Bray–Curtis dissimilarity and (**d**) Jaccard distance between CIS patients with high-risk PFO and without PFO.

Alpha and beta diversities in each sample were compared between patients with high-risk PFO and without PFO to evaluate the characteristics of the oral microbiota associated with CIS. There was no significant difference in alpha diversity between the two groups (Shannon index, *p* = 0.642; Inverse Simpson index, *p* = 0.460) (Fig. 2a,b, and Table S1). No significant differences were found in the microbial composition (beta diversity) between patients with high-risk PFO and without PFO groups (R^2^ = 0.007, F = 0.727, *p* = 0.693, Table S2). To further illustrate the microbial composition, PCoA was used to examine the extent of similarity of the oral microbial communities based on Bray–Curtis dissimilarity (Fig. 2c) and Jaccard distance (Fig. 2d).

**Fig. 3:**
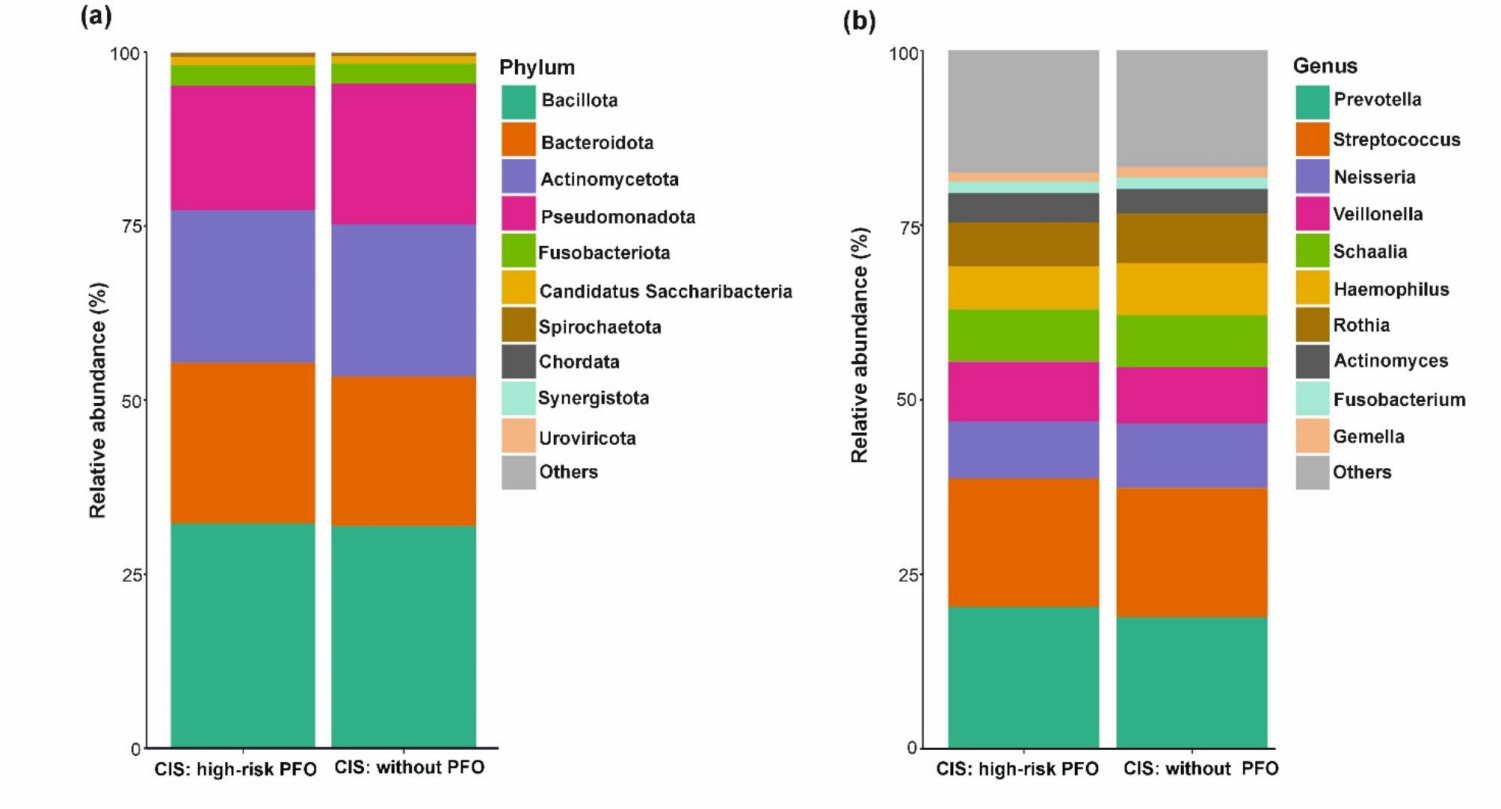
Relative abundance of oral microbiome in CIS patients with high-risk PFO and without PFO. Stacked bar chart illustrating the mean relative abundance of microbial taxa at the (**a**) phylum and (**b**) genus levels in CIS patients with high-risk PFO and without PFO.

**Fig. 4:**
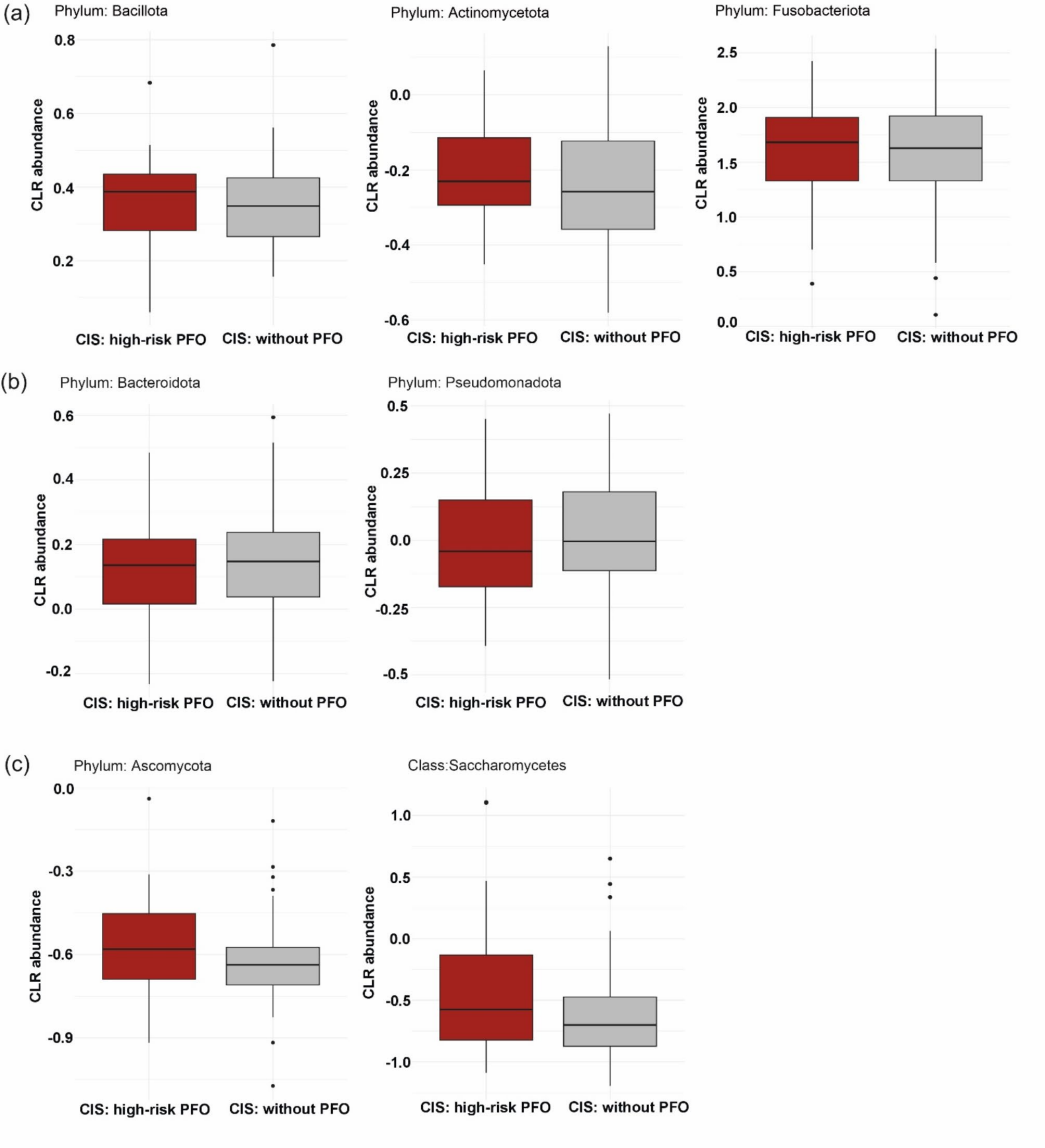
Taxonomic composition of the oral microbiome in CIS patients with and without high-risk PFO. Boxplots showing the abundance of the top phyla that were (**a**) higher and (**b**) lower in CIS patients with high-risk PFO than in those without PFO. (**c**) Taxa significantly different between CIS patients with high-risk PFO and those without PFO.

To visualize differences in oral microbiome composition between patients with and without high-risk PFO, bar plots displaying the relative abundance of taxa at the phylum and genus levels are shown (Fig. 3). At the phylum level, *Bacillota*, *Actinomycetota*, and *Fusobacteriota* were more abundant (Fig. 4a), whereas *Bacteroidota* and *Pseudomonadota* were less abundant in CIS patients with high-risk PFO than in those without PFO (Fig. 4b); however, these differences were not statistically significant. At the phylum level, only *Ascomycota* was positively associated with CIS patients with high-risk PFO (adjusted for age, hypertension, and heavy alcohol use; *q* = 0.039). At the class level, *Saccharomycetes* was significantly more abundant in high-risk PFO patients (*q* = 0.047) (Fig. 4c, Table S3). None of the genera or species were statistically significant in the adjusted model.

### Microbiome diversity and composition in CIS patients and controls with high-risk PFO

We next compared the differences in the oral microbiome between CIS patients with high-risk PFO (n = 52) and stroke-free controls with high-risk PFO (n = 16). Clinical characteristics of both groups are shown in Table S4. There were no significant differences in gender distribution, age, BMI, smoking history, heavy alcohol use, abdominal obesity, dentine caries lesions, periodontitis, mucosal lesions, or regular dentist check-ups between patients and controls. A total of 17.3% of patients and no controls used antibiotics in the past 3 months; this was not statistically significant (*p* = 0.076). However, there was a significant difference in hypertension; 25% of controls and 3.8% of patients had hypertension (*p* = 0.010, Table S4). No major differences were observed in cardiac and aortic examinations between patients and controls (Table S5). The average ejection fraction was similar between patients and controls (patients: 64.6 ± 7.6%, controls: 65.5 ± 7.5%, *p* = 0.214). Atherosclerosis in the ascending aorta and aortic arch was predominantly absent in both groups, with no significant differences (Table S5).

**Fig. 5:**
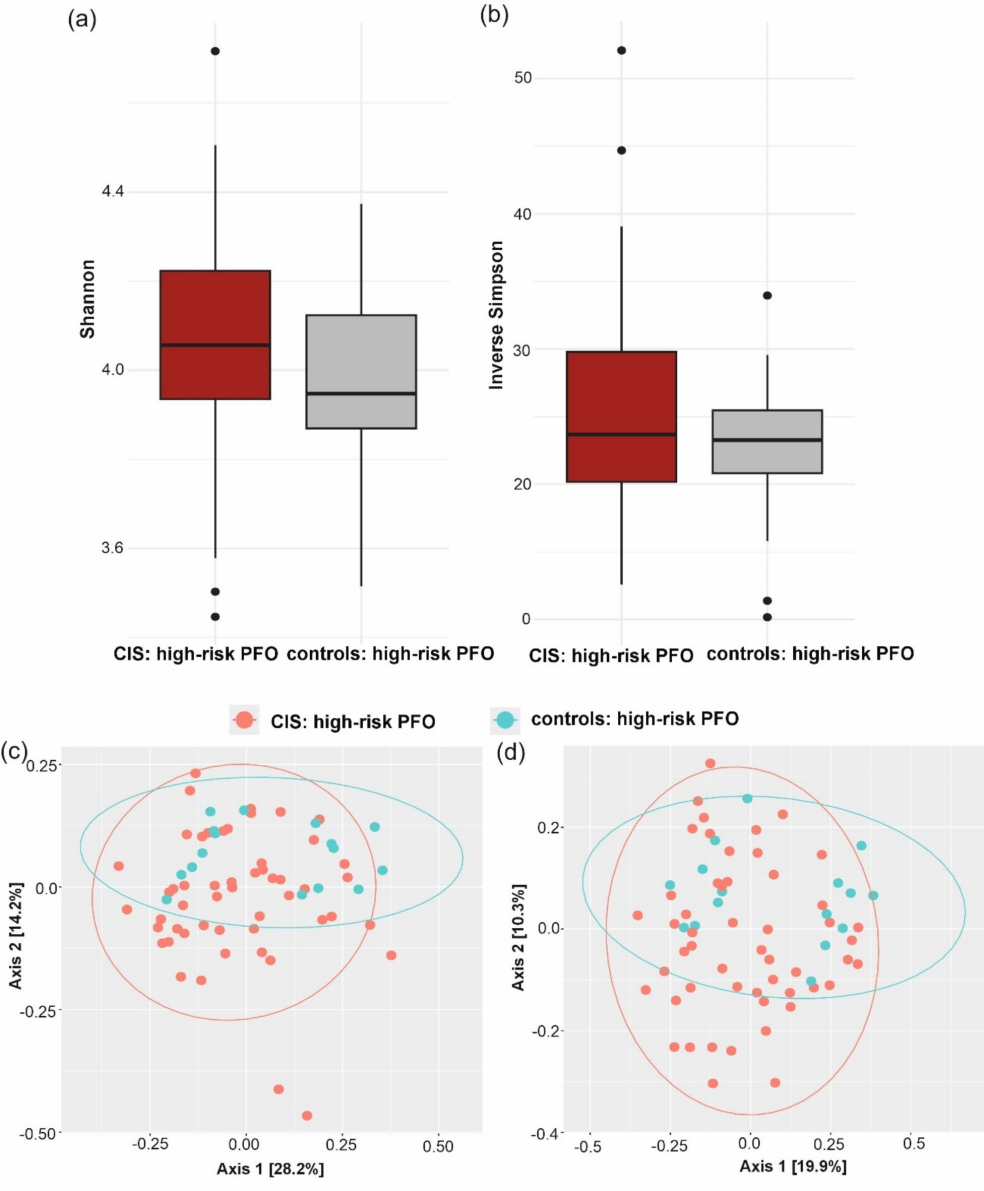
Oral microbiome diversity in CIS patients and controls with high-risk PFO. Alpha diversity measurements using the (**a**) Shannon and (**b**) Inverse Simpson index between CIS patients and controls with high-risk PFO. PCoA plot of beta diversity of the oral microbiome based on (**c**) Bray–Curtis dissimilarity and (**d**) Jaccard distance between CIS patients and controls with high-risk PFO.

**Fig. 6:**
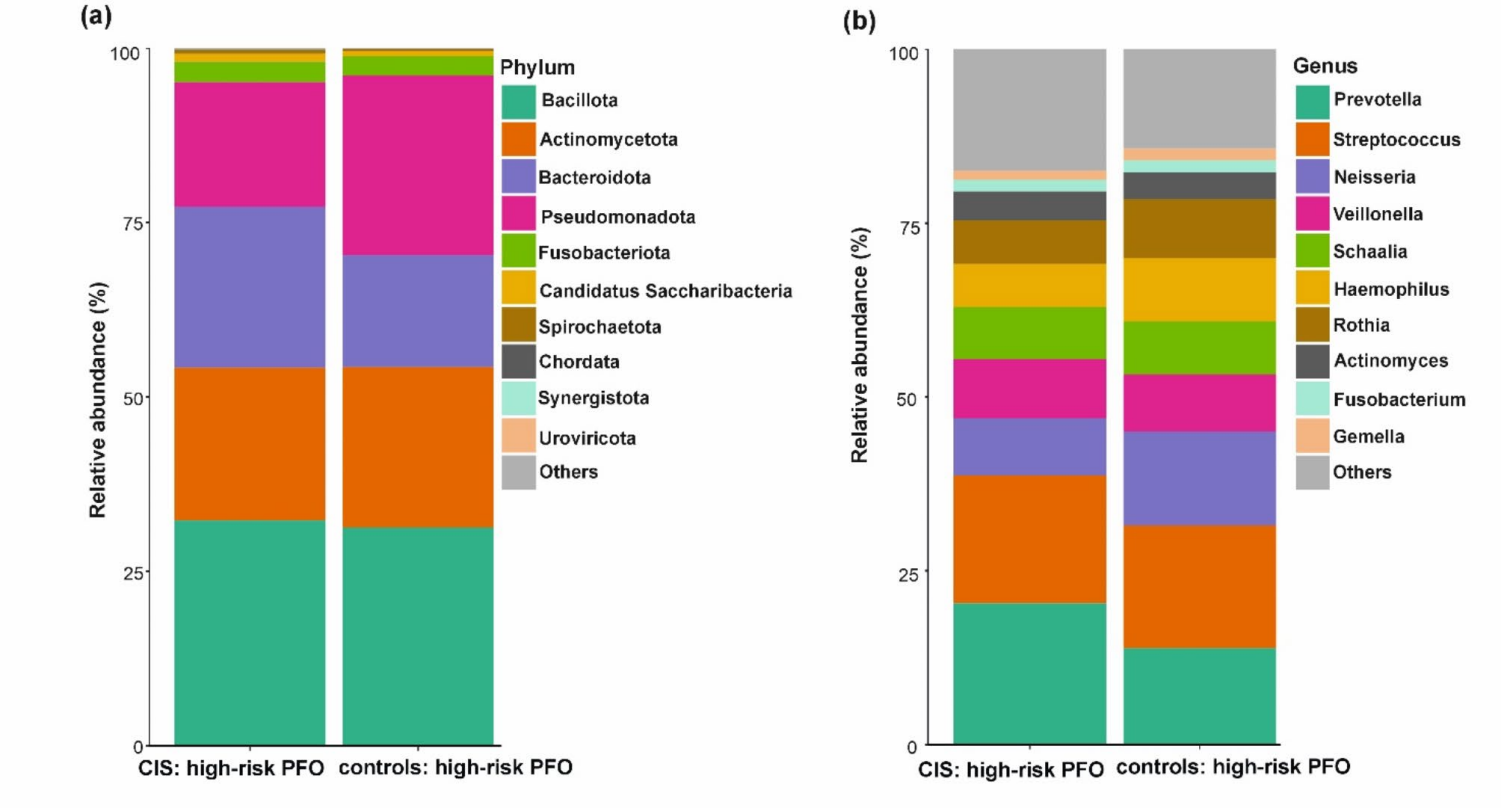
Relative abundance of phyla and genera in the oral microbiome in CIS patients and controls with high-risk PFO. Stacked bar chart illustrating the mean relative abundance of major microbial taxa at the (**a**) phylum and (**b**) genus levels in CIS patients with high-risk PFO and controls with high-risk PFO.

**Fig. 7:**
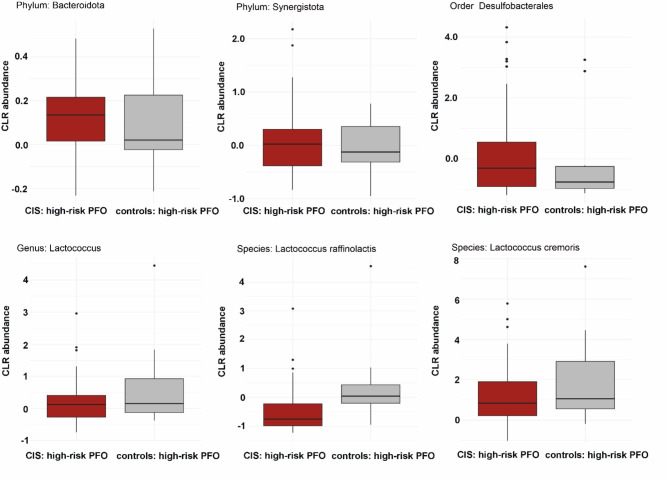
Taxonomic composition of the oral microbiome in CIS patients and controls with high-risk PFO. Boxplots showing the microbial taxa that exhibit significant differences between patients and controls with high-risk PFO.

There was no significant difference in alpha diversity between patients and controls with high-risk PFO (Shannon index, *p* = 0.197; Inverse Simpson index, *p* = 0.196) (Fig. 5a,b, and Table S6). Beta diversity analysis revealed a significant difference in microbiome composition between patients and controls with high-risk PFO (R^2^ = 0.031, F = 2.108, *p* = 0.032, Fig. 5c,d, Table S7). Bar plots showing the relative abundance of major taxa at the phylum and genus levels revealed differences in oral microbiome composition between patients and controls with high-risk PFO (Fig. 6). We found six taxa (two phyla, one order, one genus, and two species) that were significantly different between the patients and controls with high-risk PFO in the adjusted model (adjusted for hypertension). At the phylum level, *Bacteroidota* and *Synergistota* were higher in patients. Similarly, the order *Desulfobacterales* (members of the *Pseudomonadota* phylum) was significantly more abundant in patients. Conversely, the genus *Lactococcus* and species *Lactococcus raffinolactis* and *Lactococcus cremoris* were less abundant in patients than in controls (*q* < 0.05) (Fig. 7, Table S8).

## Discussion

In this study, we compared the oral microbiome in young-onset CIS patients with high-risk PFO to that of (1) young-onset CIS patients without PFO and (2) controls with high-risk PFO to investigate the relationship between oral microbiome dysbiosis and high-risk PFO. Our findings revealed that although no significant differences were observed in alpha or beta diversity between CIS patients with and without high-risk PFO, notable differences were found at various taxonomic levels. Our findings showed that *Ascomycota* was the major phylum associated with CIS patients with high-risk PFO. We identified six taxa that exhibited differential abundance between patients and controls with high-risk PFO, highlighting the significant interplay between oral microbiome composition and CIS. To the best of our knowledge, this is the first study to examine the oral microbiota and PFO in patients with CIS.

The relationship between PFO and CIS has been previously debated^28^, and knowledge of causality has increased only relatively recently after the completion of randomized clinical trials^4^. Larger PFOs are linked to an increased risk of stroke, especially when a large shunt or an atrial septal aneurysm is present^4^. The traditional mechanism connecting PFO to CIS involves paradoxical embolism, where venous blood clot bypasses pulmonary filtration and enters cerebral circulation^29^. Poor oral health, particularly periodontal disease, can lead to bacteremia, in which bacteria enter the bloodstream, increasing inflammation and the risk of clot formation. Recent surgery or use of oral contraceptives (or both), which are known to increase blood hypercoagulability, contribute to the risk of ischemic stroke^30^. Our previous findings suggest that this risk is particularly pronounced in individuals with a PFO, as evidenced by a strong association between CIS and invasive dental treatments in this population^18^.

The present study found that the abundances of the phyla *Ascomycota* and class *Saccharomycetes* were significantly higher in patients with high-risk PFO than without PFO. Similarly, the abundances of the phyla *Bacteroidota* and *Synergistota* were significantly higher in CIS patients than in the control group. Dysbiosis of the oral microbiota, especially increased proportions of Gram-negative species^31^, may contribute to an increased risk of thrombus formation, particularly in the venous system, potentially influencing the development of high-risk PFOs and subsequent thromboembolic events. Furthermore, they support the paradoxical embolism mechanism, indicating that oral microbial dysbiosis may directly contribute to PFO-related CIS through bacteraemia. This effect is further influenced by chronic inflammatory conditions, such as periodontitis, which can independently increase stroke risk by promoting systemic inflammation and bacteraemia^32,33^. However, it is important to note that our study lacked direct biomarker evidence for thrombus formation, coagulation, or inflammation to conclusively support this hypothesis. Future studies incorporating these biomarkers should provide stronger insights into the relationship between oral dysbiosis, thrombus formation, and CIS risk.

Recent studies have highlighted the putative importance of the oral fungal microbiome (mycobiome) in oral and systemic health. Oral mycobiome comprises an estimated 100 species^34^ but their association with stroke is seldom studied. In this study, we observed that *Ascomycota* (phylum) and *Saccharomycetes* (class) were the two taxa associating positively with high-risk PFO in patients. *Ascomycota* is the largest and most species-rich phylum in the fungal kingdom and is the predominant phylum in the oral cavity^35^^.^ Some genera in the phylum such as *Aspergillus* and *Candida*, which include several pathogenic species, are associated with stroke^36^. To our knowledge, these fungal taxa have not been previously reported in high-risk PFO-related CIS, and their mechanisms affecting PFO-related CIS and patient prognosis remain unclear, warranting further investigations.

In this study, we observed that at the phylum level, *Bacteroidota* and *Synergistota* were higher in patients than controls. *Bacteroidota*, one of the most prominent and abundant phylum of Gram-negative bacteria and predominant in the oral cavity, has been linked to immune dysregulation and systemic diseases through mechanisms such as glycoprotein secretion, short-chain fatty acid imbalance, and toxin production^37^. A previous animal study found that stroke induces gut microbiota dysbiosis, characterized by reduced species diversity and *Bacteroidota* overgrowth^38^. Phylum *Synergistota* comprises anaerobic bacteria with Gram-negative staining^39^. *Synergistota* is also higher in the oral cavity of patients with periodontitis^40,41^, suggesting that this phylum is disease-associated.

Compared with patients, controls in our study were enriched in the genus *Lactococcus*, including *L. raffinolactis* and *L. cremoris*. The genus *Lactococcus* was first proposed by Schleifer et al^42^ in 1985 and comprises Gram-positive, facultative anaerobic lactic acid bacteria (LAB) that ferment lactose to produce lactic acid. *Lactococcus* spp. play a significant role in oral health^43^. A recent study on oral microbiota showed that the abundance of LAB, including *Lactococcus*, was lower in the saliva of oral lichen planus cases and negatively correlated with disease severity^44^. *L. raffinolactis*, formerly known as *Streptococcus raffinolactis*, is a LAB capable of metabolizing vitamin B3 and fermenting α-galactosides, such as melibiose and raffinose^45^. *L. cremoris* is another important species within the *Lactococcus* genus, widely recognized for its significant role in the dairy industry^46^. However, its potential health benefits in the oral cavity and role in major oral and systemic diseases remain unclear. Studies have highlighted that certain species within the *Lactococcus* genus, including *L. lactis*, exhibit a range of beneficial properties, such as antimicrobial, anti-halitosis, anti-inflammatory, and inhibitory activities against periodontopathogens^47^. Additionally, *L. lactis* can adhere to dental surfaces and inhibit the growth of cariogenic bacteria such as *Streptococcus mutans*^48^. We are unaware of the exact mechanism and significance of dysbiosis in each microbial taxa, but our study findings can be used in basic research on the oral-gut-brain interaction.

Although this study provides valuable insights, there are certain limitations to consider. The present findings support that these identified microbes are associated with CIS patients who have high-risk PFO. However, it remains unclear whether these changes are a key cause of CIS related to high-risk PFO, as the cross-sectional design limits the ability to infer causation between oral microbiome dysbiosis and stroke risk. Additionally, the relatively small sample size may limit the generalizability of our findings. Saliva samples in this study were collected only once. Given that variations in the oral microbiome may occur in the period following a stroke, future research should examine microbiome changes at multiple intervals after stroke to gain a more detailed understanding of their relationship with cerebrovascular health. To better understand the dynamic changes in oral microbiota abundance, multiple saliva samples should be collected during treatment, which may help assess the potential of oral microbiota as a therapeutic target for high-risk PFO-related CIS.

## Conclusion

Our study revealed a novel association between oral microbiome dysbiosis and high-risk PFO in patients with CIS. We found changes in the oral microbial composition among CIS patients with and without high-risk PFO and when compared with controls, suggesting that these alterations may contribute to CIS risk through mechanisms such as paradoxical embolism associated with initial venous thrombosis, systemic inflammation, and bacteraemia. While these findings are promising, further research is needed to explore how oral microbiome changes affect stroke risk over time and to evaluate targeted preventive interventions.

## Electronic supplementary material

Below is the link to the electronic supplementary material.

Supplementary Material 1

## Data Availability

Sequence data that support the findings of this study have been deposited in the European Genome-Phenome Archive (accession no: EGAS00001007505).
