# Supplementary material for: Oral microbiome dysbiosis in cryptogenic ischemic stroke patients with high-risk patent foramen ovale
Source: Sci Rep. 2025 Apr 4;15:11535. doi: 10.1038/s41598-025-95728-x (PMC11971282; doi:10.1038/s41598-025-95728-x)
Supplement: Supplementary file 1 — Supplementary Material 1 [file 41598_2025_95728_MOESM1_ESM.pdf]

Oral microbiome dysbiosis in cryptogenic ischemic stroke patients with high-risk patent foramen ovale

Supplemental material

Table S1. Differences in alpha diversity between CIS patients with high-risk PFO and without PFO.

	CIS patients with high-risk PFO (n=52)	CIS patients without PFO (n=52)	Pr(>F)
Shannon index	4.07 (0.26)	4.05 (0.25)	0.642
Inverse Simpson index	25.21 (8.1)	24.12 (6.97)	0.460

Table S2. Permutational Analysis of Variance (PERMANOVA) at the species level taxa for the relative abundance features among CIS patients with high-risk PFO and without PFO.

	Df	SumOfSqs	R^2	F	Pr(>F)
patients with high-risk PFO vs without PFO	1	0.080	0.007	0.727	0.693
Residual	102	11.274	0.993	NA	NA
Total	103	11.355	1.000	NA	NA

Table S3. Differential abundance analysis showing the list of significantly different taxa between CIS patients with high-risk PFO and those without PFO. The model is adjusted for age, hypertension, and heavy alcohol use.

Taxa	Enriched group	baseMean	log2FoldChange	lfcSE	stat	pvalue	padj
Ascomycota	CIS patients with high-risk PFO	11.145	-0.6921	0.218	-3.174	0.0015	0.039
Saccharomycetes	CIS patients with high-risk PFO	5.365	-1.47651	0.444	-3.322	0.0008	0.047

Table S4. Characteristics of included CIS patients with high-risk PFO and stroke-free controls with high-risk PFO.

		**patients with high-risk PFO (n=52)**	**controls with high-risk PFO (n=16)**	***p*-value**
Gender	male	36 (69.2)	8 (50)	0.162
	female	16 (30.8)	8 (50)	
Mean age (SD), years		41.01 (6.5)	37.7 (8.1)	0.140
BMI (SD), kg/m^2		26.8 (4.1)	25.1 (2.7)	0.106
Smoking ever	yes	21 (40.4)	8 (50)	0.500
	no	31 (59.6)	8 (50)	
Heavy alcohol use	yes	7 (13.5)	3 (18.8)	0.604
	no	45 (86.5)	13 (81.3)	
Abdominal obesity	yes	25 (48.1)	5 (31.3)	0.239
	no	27 (51.9)	11 (68.8)	
Hypertension	yes	2 (3.8)	4 (25.0)	**0.010**
	no	50 (96.2)	12 (75.0)	
Antibiotics (preceding 3 months)	yes	9 (17.3)	0	0.076
	no	43 (82.7)	16 (100)	
Caries	yes	21 (40.4)	3 (18.8)	0.116
	no	31 (59.6)	13 (81.3)	
Periodontitis#	yes	11 (21.2)	4 (25.0)	0.709
	no	39 (75)	11 (68.8)	
Mucosal lesions and changes*	yes	13 (25)	5 (31.3)	0.623
	no	39 (75)	11 (68.8)	
Regular dentist check-ups#	yes	27 (51.9)	5 (31.3)	0.239
	no	25 (48.1)	11 (68.8)	
Bleeding on probing, mean (SD)		41.1 (14.9)	38.9 (14.2)	0.606
Number of missing teeth				0.993
0		36 (69.2)	11 (68.8)	
1-2		6 (11.5)	2 (12.5)	
3-5		9 (17.3)	3 (18.8)	
>5		1 (1.9)	0	
Stroke severity on admission, NIHSS score*				
no symptoms		13 (25)	-	-
mild		32 (61.5)		
moderate		6 (11.5)		
severe		1 (1.9)		

BMI, Body mass index; SD, standard deviation; Abdominal obesity; men ≥ 0.9, women ≥ 0.85; *NIHSS score: 0 (no scorable symptoms on admission), 1-4 (mild symptoms), 5-9 (moderate), and ≥10 (severe). Missing data: #periodontitis n=4, # regular dentist check-ups n=1, * Mucosal lesions and changes n=1

Table S5. Cardiac and aortic examinations and findings among the included patients with high-risk PFO and **stroke-free controls with high-risk PFO**

		**patients with high-risk PFO (n=52)**	**controls with high-risk PFO (n=16)**	***p*-value**
Transthoracic echocardiography performed	yes	52 (100)	15 (93.8)	0.071
	no	0	1 (6.3)	
Transesophageal echocardiography performed	yes	51 (98.1)	14 (87.5)	0.074
	no	1 (1.9)	2 (12.5)	
TCD-BS performed	yes	50 (96.2)	16 (100)	0.429
	no	2 (3.8)	0	
Ejection fraction (%)		64.6 (7.6)	65.5 (7.5)	0.214
PFO found by echocardiography	yes	46 (88.5)	12 (75.0)	0.781
	no	6 (11.5)	2 (12.5)	
Right-to-left shunt (PFO) found by TCD-BS$	yes	48 (92.3)	16 (100)	0.420
	no	2 (3.8)	0	
Maximum volume of interatrial shunt in echo*				0.053
small		6 (11.5)	4 (25.0)	
moderate		10 (19.2)	3 (18.8)	
severe		30 (57.7)	4 (25.0)	
Maximum volume of right-to- left shunt in TCD-BS*#				0.141
small		4 (7.7)	0	
moderate		2 (3.8)	0	
severe		42 (80.8)	16 (100)	
Atrial septal defect	yes	1 (1.9)	-	0.604
	no	51 (98.1)	14 (87.5)	
Atrial septal aneurysm#	yes	6 (11.5)	-	0.181
	no	45 (86.5)	14 (87.5)	
Atherosclerosis in ascending aortaµ	yes	-	-	1.000
	no	50 (96.2)	12 (75.0)	
Atherosclerosis in aortic arch†	yes	-	-	1.000
	no	48 (92.3)	12 (75.0)	
Aortic atherosclerosis£	yes		-	1.000
	no	50 (96.2)	12 (75.0)	

PFO, patent foramen ovale; TCD-BS, transcranial Doppler ultrasound bubble test. *Maximum volume of interatrial right-to-left shunt in echocardiography: small 1-9 bubbles, moderate: 10-24 bubbles, severe: ≥25 bubbles. †Based on information from both echocardiography and TCD-BS.

Table S6. Differences in alpha diversity between CIS patients with high-risk PFO and controls with high-risk PFO.

	CIS patients with high-risk PFO (n=52)	controls with high-risk PFO (n=16)	Pr(>F)
Shannon index	4.07 (0.26)	3.98 (0.23)	0.197
Inverse Simpson index	25.21 (8.1)	22.61 (6.28)	0.196

Table S7. Permutational Analysis of Variance (PERMANOVA) at the species level taxa for the relative abundance features among CIS patients with high-risk PFO and stroke-free controls with high-risk PFO.

	Df	SumOfSqs	R^2	F	Pr(>F)
patients with high-risk PFO vs controls with high-risk PFO	1	0.222	0.031	2.108	**0.032**
Residual	66	6.959	0.969	NA	NA
Total	67	7.181	1.000	NA	NA

Table S8. Differential abundance analysis showing the list of significantly different taxa between the patients and controls with high-risk PFO. The model is adjusted hypertension.

Taxa	Enriched group	baseMean	log2FoldChange	lfcSE	stat	pvalue	padj
Bacteroidota	patients	11.145	-0.46323	0.1625	-2.849	0.004	0.05
Synergistota	patients	11.145	-1.712	0.5801	-2.952	0.003	0.05
Desulfobacterales	patients	11.145	-3.652	0.955	-3.823	0.0001	0.015
Lactococcus	controls	116.52	2.939	0.5418	5.4255	5.78E-08	4.16E-05
L. raffinolactis	controls	4.568	3.220	0.6549	4.9177	8.75E-07	0.002
L. cremoris	controls	96.40	3.167	0.665	4.757	1.96E-06	0.0023
